# Sorghum DW1 positively regulates brassinosteroid signaling by inhibiting the nuclear localization of BRASSINOSTEROID INSENSITIVE 2

**DOI:** 10.1038/s41598-017-00096-w

**Published:** 2017-03-09

**Authors:** Ko Hirano, Mayuko Kawamura, Satoko Araki-Nakamura, Haruka Fujimoto, Kozue Ohmae-Shinohara, Miki Yamaguchi, Akihiro Fujii, Hiroaki Sasaki, Shigemitsu Kasuga, Takashi Sazuka

**Affiliations:** 1 Bioscience and Biotechnology Center, Nagoya University, Furou-cho, Nagoya, Aichi 464-8601 Japan; 2 Education and Research Center of Alpine Field Science, Faculty of Agriculture, Shinshu University, Minamiminowa, Nagano, 399-4598 Japan

## Abstract

Semi-dwarf traits have been widely introgressed into cereal crops to improve lodging resistance. In sorghum (*Sorghum bicolor* L. Moench), four major unlinked dwarfing genes, *Dw1-Dw4*, have been introduced to reduce plant height, and among them, *Dw3* and *Dw1* have been cloned. *Dw3* encodes a gene involved in auxin transport, whereas, *Dw1* was recently isolated and identified as a gene encoding a protein of unknown function. In this study, we show that DW1 is a novel component of brassinosteroid (BR) signaling. Sorghum possessing the mutated allele of *Dw1* (*dw1*), showed similar phenotypes to rice BR-deficient mutants, such as reduced lamina joint bending, attenuated skotomorphogenesis, and insensitivity against feedback regulation of BR-related genes. Furthermore, DW1 interacted with a negative regulator of BR signaling, BRASSINOSTEROID INSENSITIVE 2 (BIN2), and inhibited its nuclear localization, indicating that DW1 positively regulates BR signaling by inhibiting the function of BIN2. In contrast to rice and wheat breeding which used gibberellin (GA) deficiency to reduce plant height, sorghum breeding modified auxin and BR signaling. This difference may result from GA deficiency in rice and wheat does not cause deleterious side effects on plant morphology, whereas in sorghum it leads to abnormal culm bending.

## Introduction

In crop breeding, breeders have significantly changed plant stature during the selection of improved grain crops. One famous example is the introduction of a semi-dwarf trait into rice and wheat in the 1960s. Compared to normal plants, semi-dwarf plants have lower center of gravity, which increases lodging resistance and thus enables plant to sustain high grain yield. This phenomenon was later referred as the ‘green revolution’. The mechanism of semi-dwarfism that contributed to the ‘green revolution’ was the introduction of mutated alleles of gibberellin (GA) 20-oxidase (*semidwarf 1*; *sd1*) in rice^1, 2^ and DELLA (*Rht*) in wheat^3^, encoding a GA biosynthesis enzyme and a dominant repressor of GA signal transduction, respectively. Furthermore, it has also been reported that the semi-dwarfism of barley, caused by the introgression of *semi-dwarf 1 (sdw1/denso)* into cultivars grown in Europe, probably depends on a defect in an ortholog of rice *SD1* ^4–6^. Such wide usage of GA-related mutations to produce semi-dwarf plants has been possible due to a unique feature of GA. That is, GA deficiency specifically causes a decrease in plant height without deleterious side effects on other morphologies or physiologies, whereas dwarfism caused by other mechanisms often induces undesired phenotypes, such as abnormal leaf structure, abnormal internode elongation, and stunted seeds^7–10^. Therefore, it is rare that mutations involved in mechanisms other than GA deficiency were used to improve lodging resistance in crop breeding. One rare exception is a semi-dwarf barley mutant containing *semi-brachytic 1* (*uzu*), a weak allele of the brassinosteroid (BR) receptor, which is grown in a limited region of East Asia, including areas of Japan, Korea, and China^11^. Recently, Dockter and Hanssen^12^ reported that some barley (*Hordeum vulgare*) mutants defective in BR synthesizing genes, such as genes encoding Δ5-sterol-Δ24-reductase (*HvDIMINUTO)*, C-23 α-hydroxylase (*HvCPD)*, and BR-6-oxidase (*HvBRD*) also reduce plant height, but their contributions to breeding are very limited. Except for these barley mutants, there has not been a report, to our knowledge, that BR-related mutations have been used to affect plant height in cereal crops.

However, the situation in sorghum (*Sorghum bicolor* L. Moench) seems to differ from other cereal crops in terms of lodging-resistant breeding. Sorghum is the fifth most important cereal crop, and its grain is the dietary staple for more than 500 million people^13^. In sorghum breeding, mutations in four major unlinked dwarfing genes, *Dw1-Dw4*, have been combined to reduce plant height to increase lodging resistance and improve mechanized harvesting^14^. Among these dwarfing genes, *Dw3* and *Dw1* have been cloned, but both are unrelated to GA. *Dw3* is orthologous to maize *Brachytic2*, which encodes a gene involved in auxin transport^15^. *Dw1* was recently isolated through quantitative trait locus (QTL) analysis and encodes an uncharacterized protein, whose homologs are widely found in monocots and dicots including *Arabidopsis* ^16, 17^. A histological analysis of a SIL-05 near-isogenic line in which *Dw1* is replaced by the mutant allele *dw1* [Near isogenic line-*dw1* (NIL-*dw1*)] revealed that the longitudinal parenchymal cell lengths of the internode were almost the same between NIL-*dw1* and SIL-05 (*Dw1*), while the number of cells per internode was significantly reduced in NIL-*dw1*, demonstrating that *dw1* reduces the cell proliferation activity in the internodes^17^. In accordance, knockdown and T-DNA insertion plants of its orthologous genes in rice and *Arabidopsis*, respectively, showed similar semi-dwarfism^17^, indicating that sorghum *Dw1* and its orthologs are possibly involved in a novel mechanism to regulate plant size. From physiological perspective, it seems reasonable that sorghum does not use GA deficiency, but instead uses other mechanisms for semi-dwarfism, because in contrast to other cereals, GA deficiency in sorghum induces culm bending, which inevitably causes abnormal plant architecture^18^.

In this study, we characterized the molecular function of DW1 and revealed that *Dw1* encodes a novel component of BR signaling. BRs are plant steroid hormones that regulate multiple aspects of growth and development, including cell elongation, cell division, photomorphogenesis, and organ formation^19–21^. We suggest that DW1 acts as a positive modulator of BR signaling through inhibiting the function of a negative regulator of BR signaling, BRASSINOSTEROID INSENSITIVE2 (BIN2).

## Results

### DW1 is involved in BR signaling

**Figure 1:**
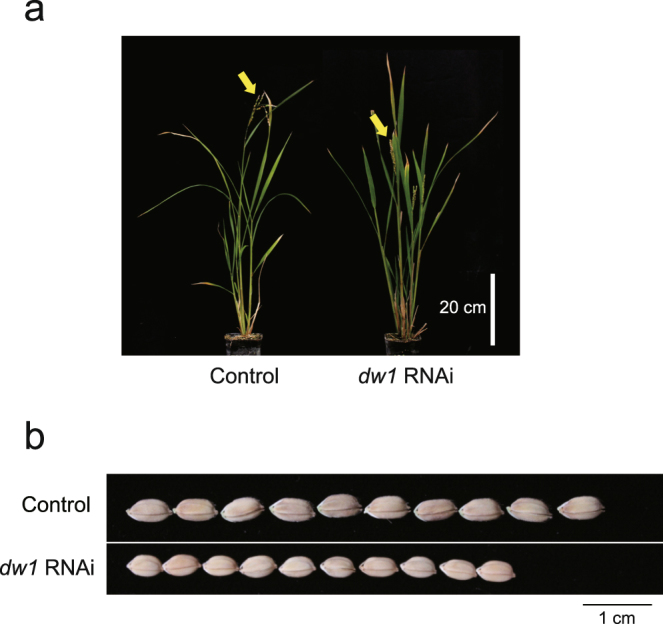
Phenotypes of *Os01g01390* and *Os03g16400* rice RNAi plant (*dw1* RNAi). (**a**) Plant stature of *dw1* RNAi plant. Vector control (Control) and *dw1* RNAi plants are presented. The positions of the panicles are shown by yellow arrows. (**b**) *dw1* RNAi plant shows short and rounded seeds. Ten seeds of vector control (Control) and RNAi plants are presented.

**Figure 2:**
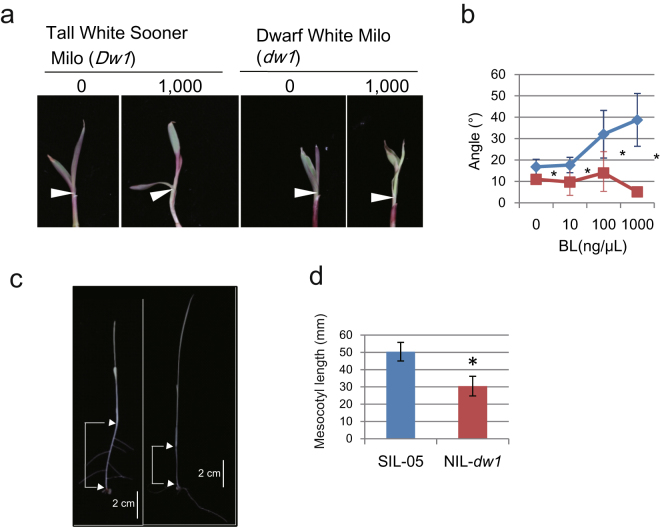
Plants carrying the *dw1* allele show defects in BL response. (**a**) Lamina joint bending test. Arrowheads indicate the position of the lamina joint. The addition of 1,000 ng/μL of BL increased the lamina joint bending of Tall White Sooner Milo (*Dw1*), but not of Dwarf White Milo (*dw1*). (**b**) Dose response of lamina joint bending in Tall White Sooner Milo (blue line) and Dwarf White Milo (red line) to BL (n = 10). Asterisks indicate *P* < 0.05 using Student’s *t*-test. (**c**) SIL-05 (left) and NIL-*dw1* (right) plants grown in complete darkness. The size of the mesocotyl is shown by square brackets. (**d**) Quantitative representation of (**c**) (n = 11). Asterisks indicate *P* < 0.05 using Student’s *t*-test.

The predicted amino acid sequence of DW1 (Sobic.009G229800) does not contain any characteristic domains or sequence and no role has been proposed for DW1 orthologs in rice or *Arabidopsis* ^17^. However, rice in which two *Dw1* orthologs (*Os01g01390* and *Os03g16400*) have been down-regulated by RNAi, showed a semi-dwarf phenotype^17^, erect leaves, and shorter and rounded seeds (Fig. 1), which are typical phenotypes of rice BR-deficient or -insensitive mutants in rice^7–10^. This prompted us to investigate the BR-related phenotypes of sorghum *dw1*. First, lamina joint bending upon BR treatment was analyzed. The lamina joint is an organ between the leaf blade and leaf sheath, which acts as a hinge to bend the leaf blade. The degree of lamina inclination in rice is known to be regulated by the synergistic action of BRs and auxin^22, 23^, although there is no report whether such a phenomenon also occurs in sorghum. The wild type sorghum, ‘Tall White Sooner Milo’, responded to an active form of BR, brassinolide (BL) (Fig. 2a), demonstrating that the lamina inclination test can also be applied to test BR sensitivity in sorghum plants. Thus, we examined the sensitivity of the mutant carrying *dw1,* ‘Dwarf White Milo’, by using this test. Tall White Sooner Milo carrying *Dw1* started to respond to an active form of BL at a concentration of 10 ng/μL (20.8 μM) and continuously increased its bending angle up to a concentration of 1000 ng/μL BL, while Dwarf White Milo (*dw1*) did not show any clear response even at a concentration of 1000 ng/μL BL (Fig. 2a,b), suggesting that DW1 is involved in BR response. The fact that Dwarf White Milo (*dw1*) did not respond to exogenously applied BL further suggests that it is not the BR synthesis, but BR signaling that is defective in *dw1* sorghum. Next, we examined the effect of DW1 on skotomorphogenesis, which is another phenomenon regulated by BR. We grew sorghum plants in complete darkness and measured the mesocotyl length (Fig. 2c,d). For this experiment, we used a NIL of SIL-05 carrying *dw1* (NIL-*dw1*)^17^ and compared it to SIL-05 (*Dw1*). SIL-05 developed an elongated mesocotyl in response to darkness, while NIL-*dw1* produced shorter mesocotyls than SIL-05 (Fig. 2c,d). This observation again demonstrates that DW1 is involved in BR response.

### Expression of BR-related genes in *dw1*

**Figure 3:**
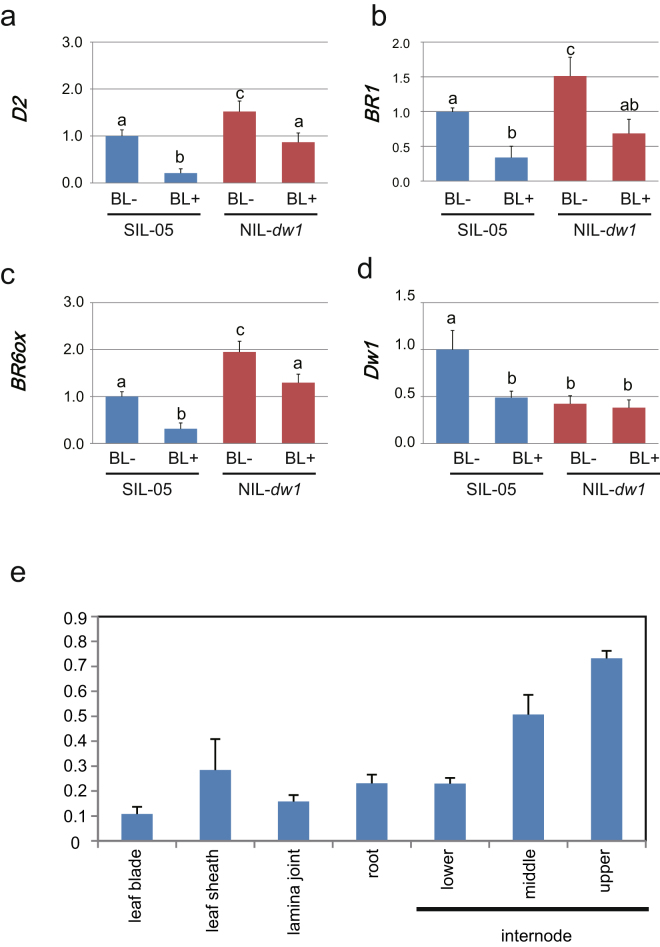
Expression of BR-related genes. (**a–d**) BL feedback regulation of *Dw1* and BR-related genes. Expression of SIL-05 (blue bars) and NIL-*dw1* (red bars) treated with or without BL are shown. Bars labeled with different letters differ significantly (Tukey–Kramer HSD test, *P* < 0.05). (**e**) Expression of *Dw1* in various organs of Tall White Milo. Expression levels are shown relative to the expression of a sorghum ubiquitin gene. More than 3 individuals were analyzed.

To further confirm that DW1 is involved in BR signaling, we examined the expression of BR-related genes in *dw1*. Activation of BR signaling in turn inhibits transcription of some BR biosynthesis and signaling genes in rice and *Arabidopsis* as a feedback mechanism to fine-tune BR response^24–26^. Sorghum BR genes, which correspond to the *ebisu dwarf* (*SbD2*), *BR 6-oxidase* (*SbBR6ox*), and *BRASSINOSTEROID INSENSITIVE 1* (*SbBRI1*) genes were identified through phylogenetic analysis (see Supplementary Figs S1–S3), and their expression was analyzed. *D2* and *BR6ox* encode enzymes for the late steps of BR biosynthesis and *BRI1* encodes a BR receptor. The corresponding genes of sorghum showed significantly higher expression in NIL-*dw1* than in its original strain, SIL-05 (Fig. 3a–c). When BL was applied to these seedlings, their expression was repressed in both plants; however, the suppressive effect of BL was milder in NIL-*dw1* than that in SIL-05 (Fig. 3a–c), indicating that BR signaling is attenuated in NIL-*dw1*. The BL treatment also repressed the expression of *Dw1* in SIL-05 but not in NIL-*dw1* (Fig. 3d), also confirming that DW1 is related to BR signaling. The reason for the reduced expression of *dw1* in NIL-*dw1* (BL−) compared to *Dw1* in SIL-05 (BL−) is unknown. Because *dw1* does not contain a mutation in its promoter region and is assumed to be a null allele caused by a premature stop codon in its coding region, the altered mRNA might have affected *dw1* expression.

### *Dw1* is highly expressed in the young internode

We next analyzed the expression of *Dw1*. Field grown Tall White Milo (*Dw1*) 1–2 weeks before the heading stage was used for the analysis. Expression of *Dw1* was detected in all of the organs analyzed, including the leaf blade, the sheath, and the root (Fig. 3e). When internodes were divided into lower, middle, and upper regions, the expression increased from the lower to the upper part of the internode. The lower part of the internodes elongate at the early vegetative stage, whereas the uppermost internode extensively elongates just prior to the heading stage. That *Dw1* showed the highest expression in the upper internode is consistent with DW1 functioning in internode elongation^16, 17^.

### DW1 inhibits nuclear localization of BIN2

**Figure 4:**
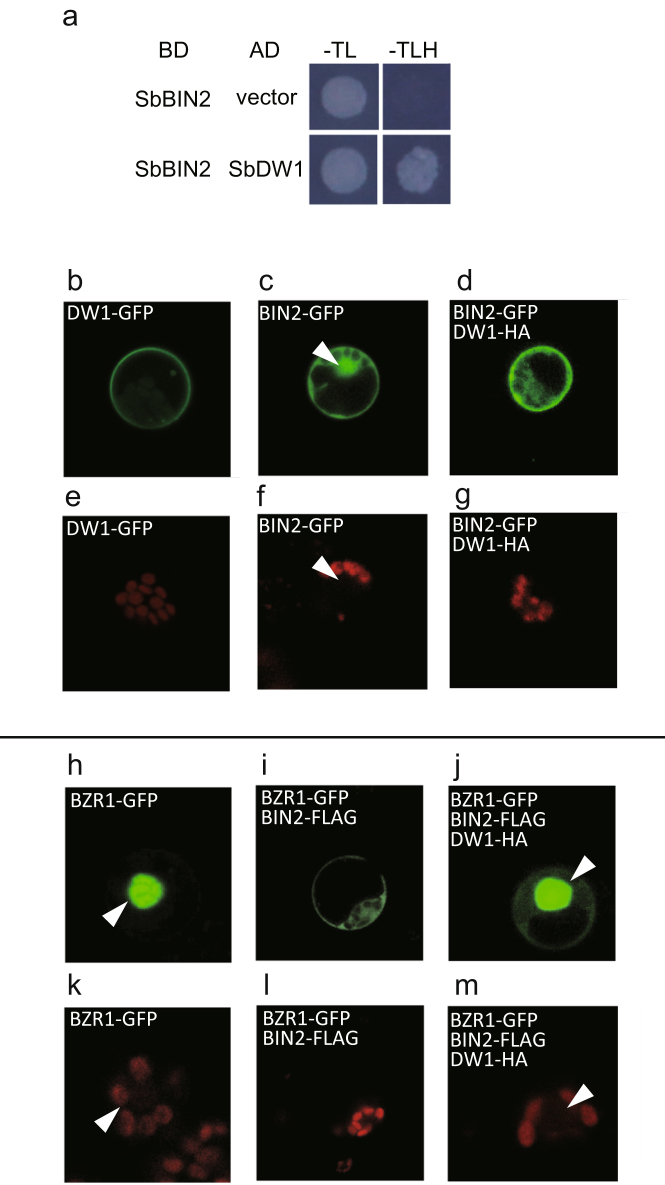
DW1 interacts with BIN2 and inhibits BIN2 from localizing to the nucleus. (**a**) Interaction of SbDW1 and SbBIN2 in a Y2H assay. The growth of yeast strain AH109 transformants is shown. SbBIN2 was used as the bait, and SbDW1 was used as the prey. −TL, synthetic complete medium lacking Trp and Leu; −TLH, synthetic complete medium lacking Trp, Leu, and His. (**b–d**, **h–j**) Transient expression of DW1, BIN2, and BZR1 in rice protoplasts. (**b**) DW1 localizes on the plasma membrane and in the cytosol. (**c**) BIN2 localizes to the nucleus and the cytosol. (**d**) Simultaneous expression of DW1 and BIN2 inhibits nuclear localization of BIN2. (**h**) BZR1 localizes to the nucleus and the cytosol. (**i**) Simultaneous expression of BZR1 and BIN2 inhibits nuclear localization of BZR1. (**j**) DW1, BIN2, and BZR1 co-expression results in BZR1 localizing to the nucleus. Rice protoplasts were transformed with either BIN2-GFP, BZR1-GFP, DW1-GFP, and co-transformed with BIN2-FLAG, DW1-HA, or BIN2-FLAG/DW1-HA. **(e–g**, **k–m**) Chloroplast autofluorescence of rice protoplasts are shown as the red channel.

Various factors that play a positive role in BR signaling interact with BIN2. Thus, we performed a yeast two-hybrid assay (Y2H) to test the physical interaction between DW1 and BIN2. For this purpose, we isolated sorghum ortholog of BIN2 (SbBIN2) (see Supplementary Fig. S4). As expected, DW1 and SbBIN2 tightly interacted in yeast cells (Fig. 4a). Similarly, rice DW1 orthologs (LOC_Os01g01390 and LOC_Os03g16400) also interacted with rice BIN2 (OsBIN2), respectively (see Supplementary Fig. S5). To further investigate the effect of the DW1-BIN2 interaction *in planta*, cellular localization of sorghum DW1, BIN2, and BRASSINAZOLE RESISTANT 1 (BZR1) were analyzed (Fig. 4b–d). The analysis was conducted in rice protoplasts because the transient expression using sorghum protoplasts did not work. When DW1 fused with Green Fluorescent Protein (GFP) was expressed, the GFP signal was localized on the plasma membrane or in the cytosol (Fig. 4b), whereas as previously reported, BIN2 was localized in the cytosol or in the nucleus (Fig. 4c, arrowhead indicates the nucleus). When both *BIN2-GFP* and *DW1-Human influenza hemagglutinin (HA)* were co-transformed, the GFP signal of BIN2 disappeared from the nucleus (Fig. 4d), suggesting that DW1 inhibits the nuclear localization of BIN2. Next, because BIN2 is known to phosphorylate BZR1 and direct BZR1 out of the nucleus, the effect of DW1 on BZR1 localization was investigated. BZR1-GFP alone localized to the nucleus and the cytosol (Fig. 4h), whereas co-transformation with *BIN2-FLAG* led the GFP signal to be restricted to the cytosol (Fig. 4i), which is consistent with previous reports that BIN2 phosphorylates and inhibits the transcriptional activity of BZR1 by directing BZR1 out of the nucleus^27, 28^. When *BZR1-GFP*, *BIN2-FLAG*, and *DW1-HA* were co-transformed, the GFP signal of BZR1 was localized to the nucleus (Fig. 4j). Similar results were also obtained when DW1, BIN2, and BZR1 of *Arabidopsis* were expressed in *Arabidopsis* protoplasts (see Supplementary Fig. S6). These observations suggest that DW1 localizes to the plasma membrane and the cytosol and interacts with BIN2 to suppress the repressive function of BIN2, allowing BZR1 to localize to the nucleus (see Discussion).

### Emergence of DW1 in the plant lineage

**Figure 5:**
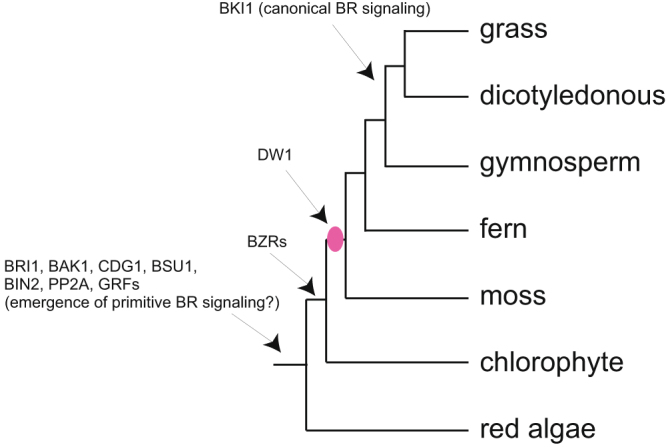
Origin of DW1 in plants. The pink circle indicates the point of possible emergence of DW1. BKI1 arose after the split of gymnosperms and angisperms, whereas the other 8 BR signaling components arose at early stages of plant evolution^29^.

**Figure 6:**
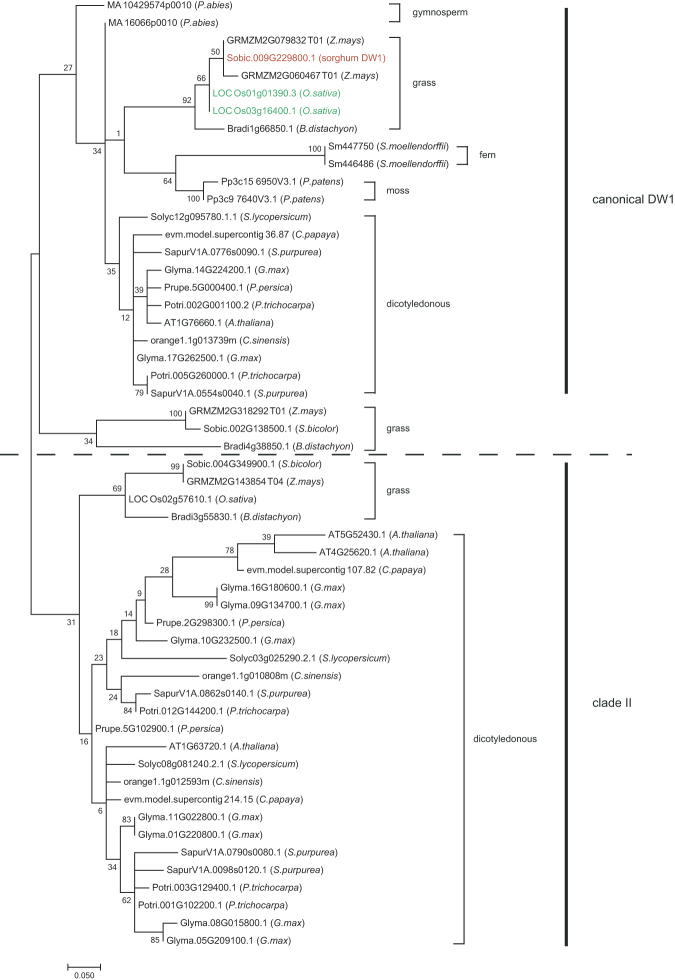
Phylogenetic analysis of DW1 homologs. A maximum likelihood tree based on the JTT model^38^ was obtained. The horizontal branch lengths are proportional to the estimated number of amino acid substitutions per residue. Bootstrap values were obtained by 100 bootstrap replicates. SbDW1 are shown in red, and rice DW1 homologs used to create the RNAi rice are shown in green.

Wang *et al*.^29^ proposed that canonical BR signaling originated after the split of gymnosperms and angiosperms. This assumption is based on BRI1 KINASE INHIBITOR 1 (BKI1), a negative regulator of BR signaling, being present only in the angiosperm lineage, while homologs or orthologs of the other 8 BR signaling components [BRI1, BRASSINOSTEROIDINSENSITIVE1-ASSOCIATED RECEPTOR KINASE1 (BAK1), CONSTITUTIVE DIFFERENTIAL GROWTH1 (CDG1), BRASSINOSTEROID INSENSITIVE1 SUPPRESSOR1 (BSU1), BIN2, BZRs, PROTEIN PHOSPHATASE 2A (PP2A), GENERAL REGULATORYFACTORS (GRFs)] are found in all the land plants and also in algae (Fig. 5). The only exception is that BZRs are missing in some of the algae species including red algae. To investigate when DW1 originated in plant evolution, we searched for the existence of DW1 homologs in 15 land plants and 5 chlorophytes, for which the whole genome sequences are available (https://phytozome.jgi.doe.gov/pz/portal.html, http://congenie.org/). Our data set includes the genome sequences of 4 grasses (*Sorghum bicolor*, *Oryza sativa*, *Brachypodium distachyon*, and *Zea mays*), 8 dicots (*Arabidopsis thaliana*, *Glycine max*, *Solanum lycopersicum*, *Prunus persica, Citrus sinensis, Carica papaya, Salix purpurea,* and *Populus trichocarpa*), 1 gymnosperm (*Picea abies*), 1 lycophyte (*Selaginella moellendorffii*, a fern), 1 bryophyte *(Physcomitrella patens*, a moss), and 5 chlorophytes (*Chlamydomonas reinhardtii, Coccomyxa subellipsoidea, Micromonas pusilla, Ostreococcus lucimarinus*, and *Volvox carteri*). Similar to those observed for 8 BR signaling components, DW1 homologs were present in all of the land plants including *S. moellendorffii* and *P. patens* with varying copy numbers of homologs in each species (see Supplementary Table S1). However, a DW1 homolog could not be found in any of the 5 chlorophyte genomes, indicating that DW1 arose in the land plant lineage after the divergence from chlorophytes. When a phylogenetic tree of DW1 homologs was constructed, two large clades were observed (Fig. 6, see also Supplementary Figs S7 and S8). One clade consisted of SbDW1 and thus we named it as the canonical DW1 clade. Within this clade, there were DW1 homologs of fern (Sm44686 and Sm447750) and moss (Pp3c9 7640v3.1 and Pp3c15 6950V3.1), whereas, in the second clade (clade II), no homologs of fern and moss were present, indicating that clade II DW1 homologs originated from canonical DW1 in seed plants. Within the canonical DW1 clade, each plant species contained either one or several homologs, suggesting that either some species lost or duplicated the *Dw1* gene homologs during evolution.

## Discussion

In this study, molecular biological experiments demonstrated that DW1 is positively involved in BR signaling. Simultaneous knockdown of two rice canonical *Dw1*s led to semi-dwarfism with erect leaves and shortened and rounded seeds (Fig. 1). Dwarf White Milo carrying *dw1* did not respond to BL treatment in a lamina joint inclination assay (Fig. 2a,b). In addition, compared to SIL-05 (Dw1), NIL-*dw1* had a shorter mesocotyl length when grown in the dark (Fig. 2c,d). We also observed that the feedback regulation of BR-related genes in SIL-05 was attenuated in NIL-*dw1* (Fig. 3a–d).

DW1 directly interacted with a negative regulator of BR signaling, BIN2 (Fig. 4a), and our transient expression analyses showed that DW1, which localizes to the plasma membrane and the cytosol, functions as a modulator of BR signaling by regulating the intracellular localization of BIN2. That is, expression of DW1 eliminated BIN2 from the nucleus, which in turn allowed nuclear localization of BZR1 (Fig. 4d,j). Up to now, three BIN2 inactivating mechanisms have been proposed. In the first mechanism, BRs enhance BIN2 degradation mediated by the 26S proteasome by an unknown mechanism^30^. Another mechanism is that direct binding of HISTONE DEACETYLASE 6 to BIN2 deacetylates BIN2, which leads to inhibition of BIN2 kinase activity and represses BIN2 function^31^. The third inactivating mechanism is similar to that proposed for DW1. OCTOPUS (OPS), a polarly localized plasma membrane-associated protein that functions in phloem differentiation, recruits BIN2 to the plasma membrane^32^. Localization of BIN2 to the plasma membrane by OPS prevents BIN2 from phosphorylating BZR1 and BES1 in the nucleus. Although OPS is specifically expressed in the phloem, it would be interesting to examine if any interaction occurs between OPS and DW1 or if they each independently function to inhibit BIN2 to the localize to the nucleus.

Our comparative genomic analysis suggests that DW1 arose in the land plant lineage after the divergence from chlorophyta (Fig. 5). This is in clear contrast to the other BR components, which are present even in algae, with the exception of BKI1 which originated after the split of gymnosperms and angiosperms^29^. It is tempting to speculate that if a primitive BR signaling lacking BKI1 exists in plants other than angiosperms, the emergence of DW1 in land plants should have enabled plants to fine-tune BR signaling through BIN2 inactivation.

To reduce plant height to aid cultivation, sorghum was bred to contain four mutant genes, *Dw1-Dw4*, whereas in rice and wheat, modifying a single gene related to GA was sufficient. Rice and wheat can repress GA signaling to a certain degree without accompanying deleterious effects on plant morphology, but in sorghum, it leads to abnormal culm bending^18^. This led to a conundrum in sorghum because reducing plant height by severely modifying other mechanisms often leads to negative side effects in plants. To avoid these side effects in sorghum, it might have been necessary to combine several mutations, each only mildly affecting different hormone signaling. A mutation in one of these four genes, *Dw1*, may cause only a mild effect on BR signaling because it is not an evolutionarily conserved component of BR signaling. Our phylogenic analysis of DW1 homologs shows that DW1 arose at a later stage in plant evolution than other BR signaling factors and therefore might have been a suitable component to mutate as it functions in fine-tuning BR signaling rather than being a central component of it.

## Methods

### Rice transformation

Construction and transformation of RNAi transgenic rice to simultaneously knockdown *Os01g01390* and *Os03g16400* were previously described^17^.

### Lamina joint inclination assay and skotomorphogenesis analysis

The lamina joint inclination assay was performed according to Fujioka *et al*.^33^. BL was dissolved in ethanol at final concentrations of 10 ng/μL, 100 ng/μL, and 1000 ng/μL. Germinated seeds were selected for uniformity of coleoptile length, transplanted onto 0.7% agar medium, and grown at 30 °C for 2 days. The seedlings were injected at the top of the lamina with 1 μL of ethanol solution containing 0, 10, 100, or 1,000 ng of BR intermediates with 5 μg indole-3-acetic acid. After incubating for 3 days, the angle between the lamina and its leaf sheath was measured^33^. There were 10 plants in each treatment. For the skotomorphogenesis analysis, NILs were germinated and grown on 0.65% agar in complete darkness at 30 °C for 2 weeks and then the lengths of mesocotyls were measured.

### RNA extraction and semi-quantitative reverse transcription polymerase chain reaction (RT-PCR) analyses

To analyze feedback regulation using semi-quantitative RT-PCR analysis, SIL-05 and NIL-*dw1* were grown on Murashige and Skoog basal salt mixture (MS) agar in a growth chamber for 2 weeks under constant light at 30 °C. For BL treatment, BL (Daiichi Fine Chemical) dissolved in 0.01% ethanol was included in the MS agar. Mock-treated plants were grown on MS agar containing 0.01% ethanol. Total RNA was isolated from whole seedlings using an RNeasy Plant Mini Kit (Qiagen). The first-strand complementary DNA (cDNA) was synthesized using an Omniscript RT Kit (Qiagen) and was used in a real-time PCR detection system (Bio-Rad CFX96). Gene expression was normalized to that of sorghum *ubiquitin* (*Sobic.001G311100*; *SbUbi*), and ratios of the expression levels in NIL-*dw1* compared to SIL-05 (set as 1.0) were calculated. Three repeats were performed for each gene, and the average values and standard deviations are shown. To analyze the global expression of *Dw1*, field grown Tall White Milo was collected before heading. Other procedures were conducted identical to those stated above. Primer sequences are listed in Supplementary Table S2.

### Yeast Two-Hybrid

To construct DNA binding domain (BD)-SbBIN2 and Activation domain (AD)-SbDW1, *SbBIN2* and *SbDW1* cDNA were PCR amplified and cloned into the *EcoR*I/*Sal*I site of pGBKT7 and *BamH*I/*Xho*I site of pGADT7, respectively. The yeast two-hybrid assay (Y2H) was performed as described previously^34^ using the BD Matchmaker Two-Hybrid System 3 (Clontech). Vector cassettes for the DNA-AD were used as negative controls, and *Saccharomyces cerevisiae* strain AH109 was used as the host. Details of the methods used for the yeast assays can be found in the manufacturer’s instructions (Yeast Protocols Handbook #PT3024-1; http://www.clontech.com/).

### Transient expression assay using *Arabidopsis* mesophyll protoplasts

Three plasmids, *Dw1-GFP*/pE2113, *BIN2-GFP*/pE2113, and *BZR1-GFP*/pE2113 were constructed for the transient assay as follows. A PCR-amplified *GFP* gene fragment was introduced into the *Spe*I/*Stu*I site of the pE2113_GW_SAS vector^35^. Then PCR-amplified protein-coding regions of *Dw1* (Sobic.009g229800), *BIN2* (At4g18710) and *BZR1* (At1g75080) were introduced into the *Xba*I/*Spe*I site of the vector in the same reading frame as *GFP*. To construct the plasmids *Dw1-HA*/pE2113 and *BIN2-FLAG*/pE2113, a synthetic DNA encoding the HA tag and a PCR-amplified FLAG fragment were introduced into *Spe*I/*Stu*I site instead of *GFP*. All of the genes were driven by the *35S* promoter, and all of the constructs were carried out using the In-Fusion HD Cloning Kit (Clontech). The plasmids were extracted using the QIAGEN Plasmid Maxi Kit (QIAGEN) and used for the transient expression assay. The sequences of the primers used for PCR amplification or oligonucleotides used in this study are listed in Supplementary Table S2. Isolation of mesophyll protoplasts and transient gene expression assays were performed according to Zhang *et al*.^36^, Wu *et al*.^37^ and Ryu *et al*.^27^. GFP fluorescence was recorded by a LSM 700 confocal microscope (Zeiss).

### Phylogenetic tree analysis

The amino acid sequence of sorghum DW1 was used as a query to screen databases (https://phytozome.jgi.doe.gov/pz/portal.html, http://congenie.org/) by tblastn and candidate genes were selected for phylogenetic analyses. The e-value threshold was set at 5.8E-07. Sequences were aligned with ClustalW (Mega 7) with default parameters, and phylogenetic trees (maximum likelihood, neighbor-joining, and maximum parsimony methods) were created by Mega 7 with default parameters. Bootstrap values were obtained by 100 bootstrap replicates.

## Electronic supplementary material

Supplementary files
