# Supplementary material for: Sorghum DW1 positively regulates brassinosteroid signaling by inhibiting the nuclear localization of BRASSINOSTEROID INSENSITIVE 2
Source: Sci Rep. 2017 Mar 9;7:126. doi: 10.1038/s41598-017-00096-w (PMC5428356; doi:10.1038/s41598-017-00096-w)
Supplement: Supplementary file 1 — Supplementary files [file 41598_2017_96_MOESM1_ESM.pdf]

Supplemental Data

Title: Sorghum DW1 positively regulates BR signaling by inhibiting the nuclear localization of BIN2

Authors: Ko Hirano, Mayuko Kawamura, Satoko Araki-Nakamura, Haruka Fujimoto, Kozue Ohmae-Shinohara, Miki Yamaguchi, Akihiro Fujii, Hiroaki Sasaki, Shigemitsu Kasuga, Takashi Sazuka

Supplementary figures: S1 to S8

Supplementary tables: S1 to S2

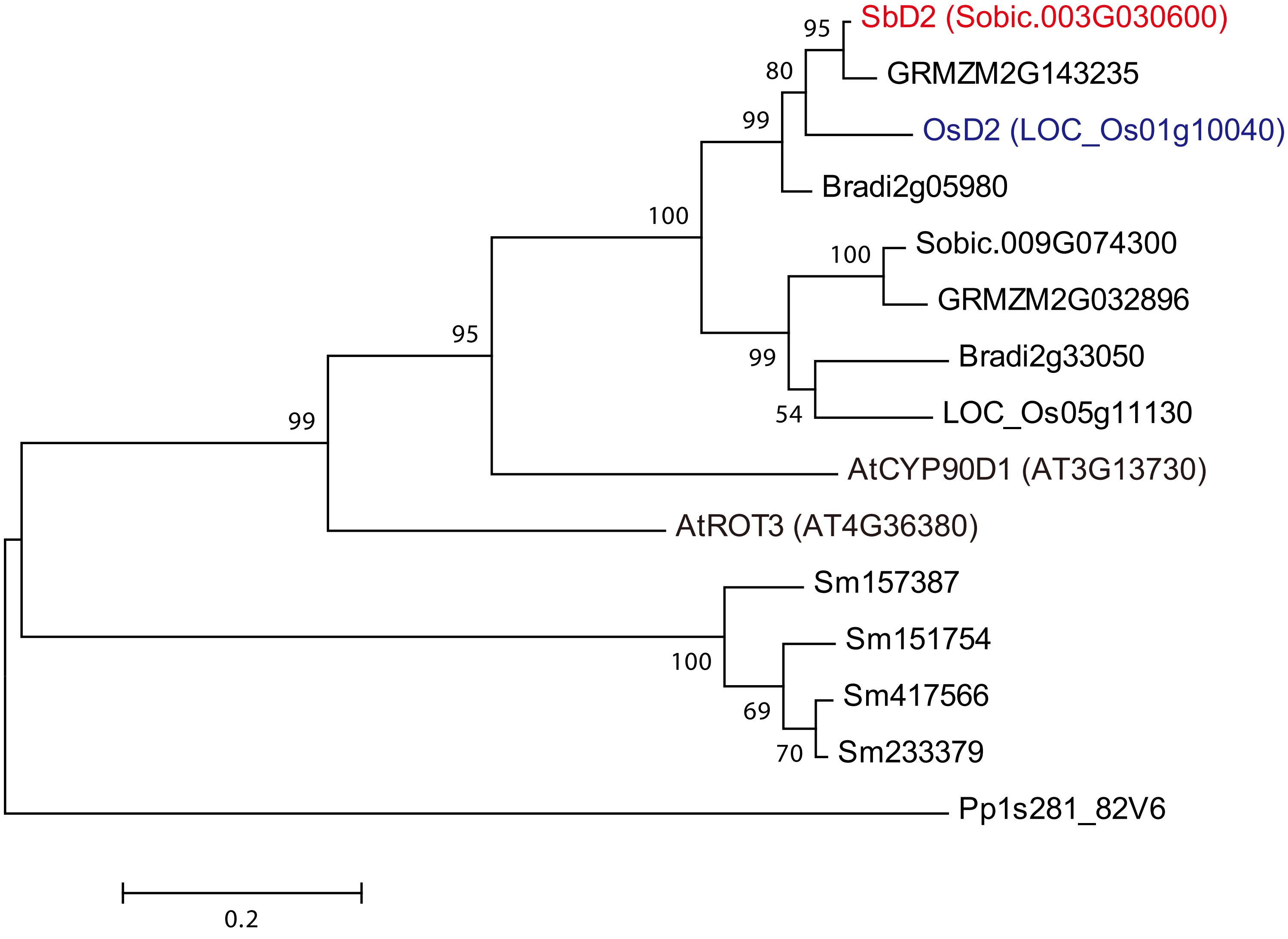

Fig. S1. Phylogenetic analysis of D2 homologs using the NJ method. SbD2 (Sobic.003G030600) and OsD2 (LOC_Os01g10040) are shown in red and blue, respectively. Bootstrap values were obtained by 100 bootstrap replicates.

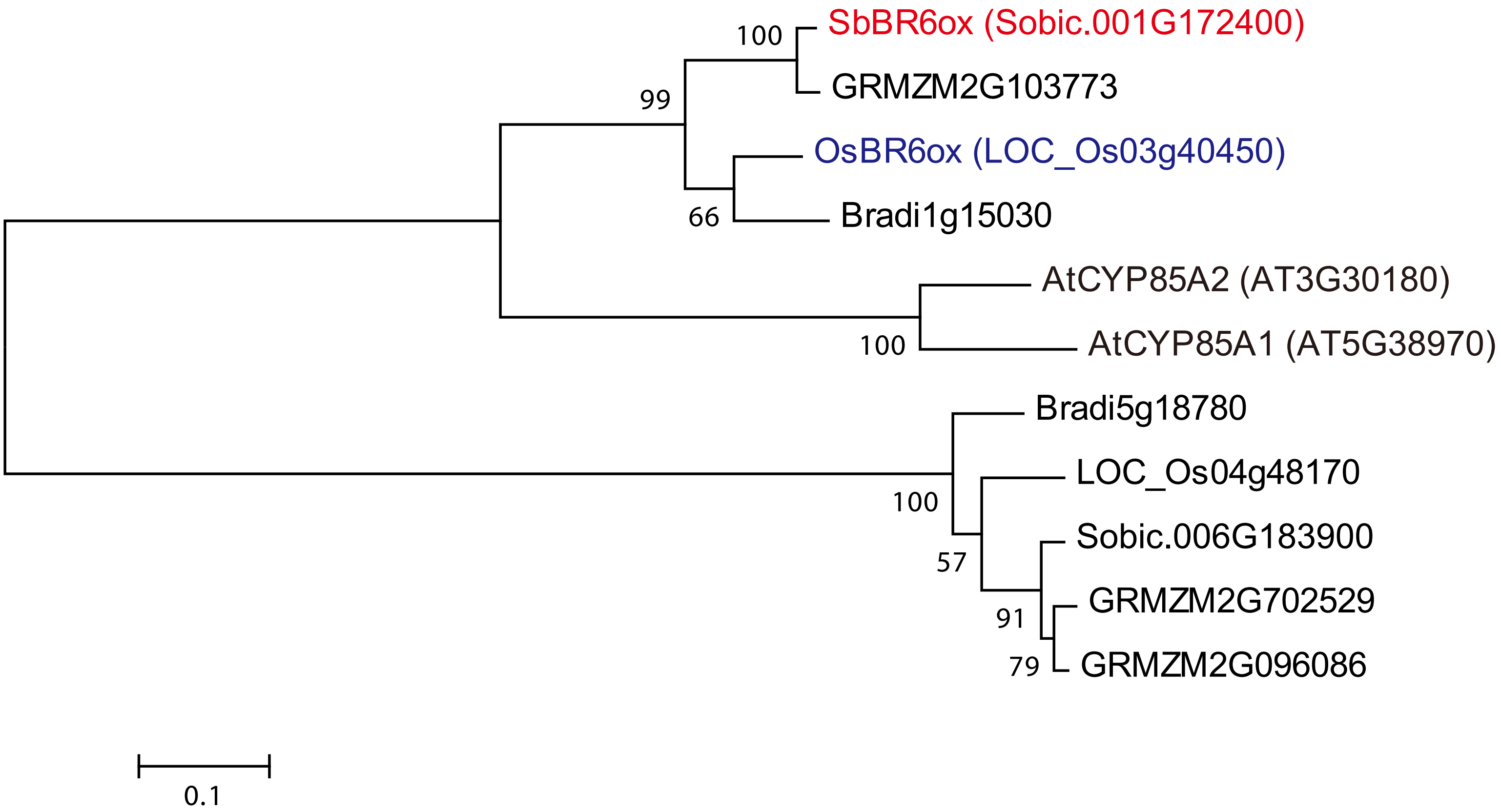

Fig. S2. Phylogenetic analysis of BR6ox homologs using the NJ method. SbBR6ox (Sobic.001G172400) and OsBR6ox (LOC_Os03g40540) are shown in red and blue, respectively. Bootstrap values were obtained by 100 bootstrap replicates.

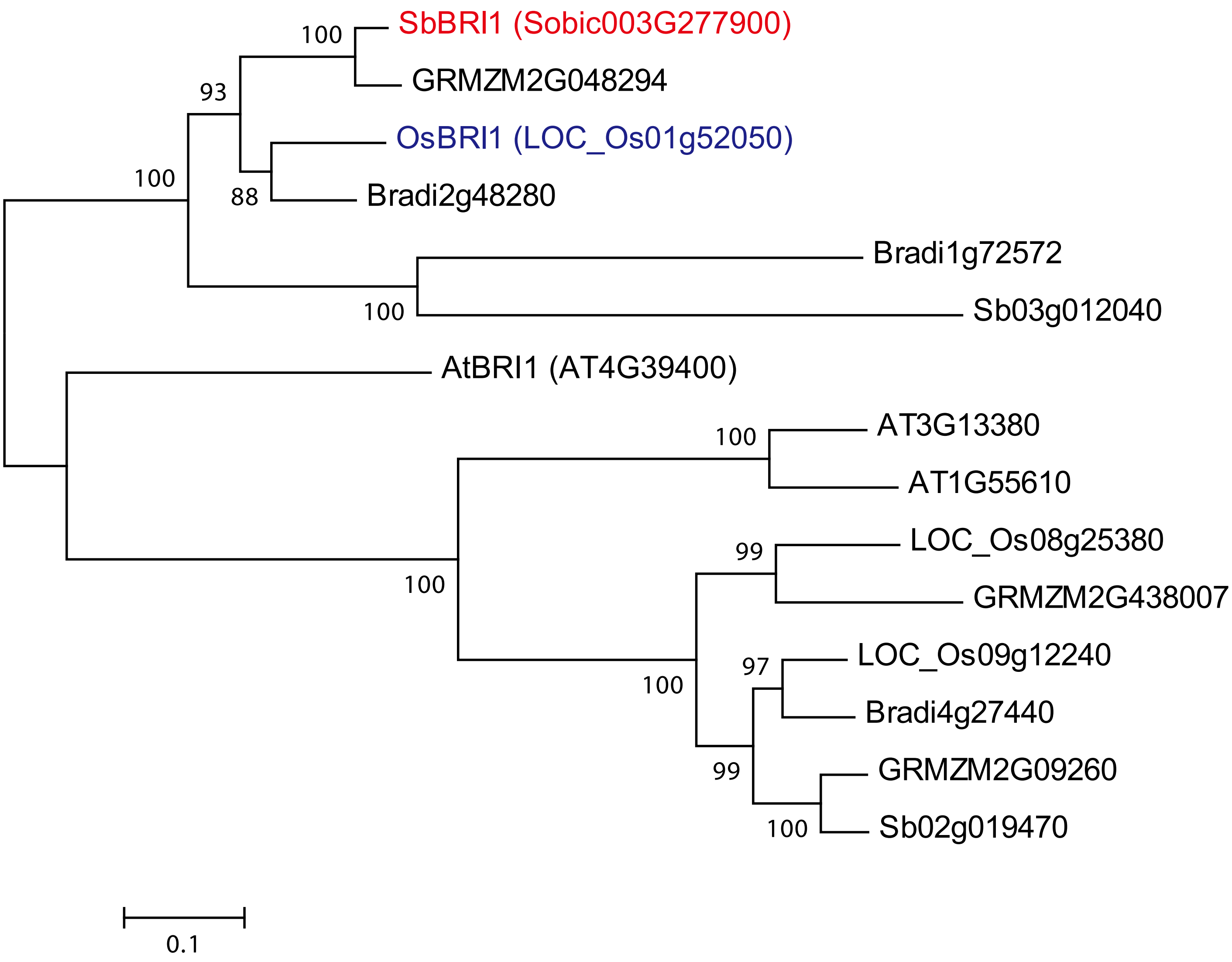

Fig. S3. Phylogenetic analysis of BRI1 homologs using the NJ method. SbBRI1 (Sobic.003G277900) and OsBRI1 (LOC_Os01g52050) are shown in red and blue, respectively. Bootstrap values were obtained by 100 bootstrap replicates.

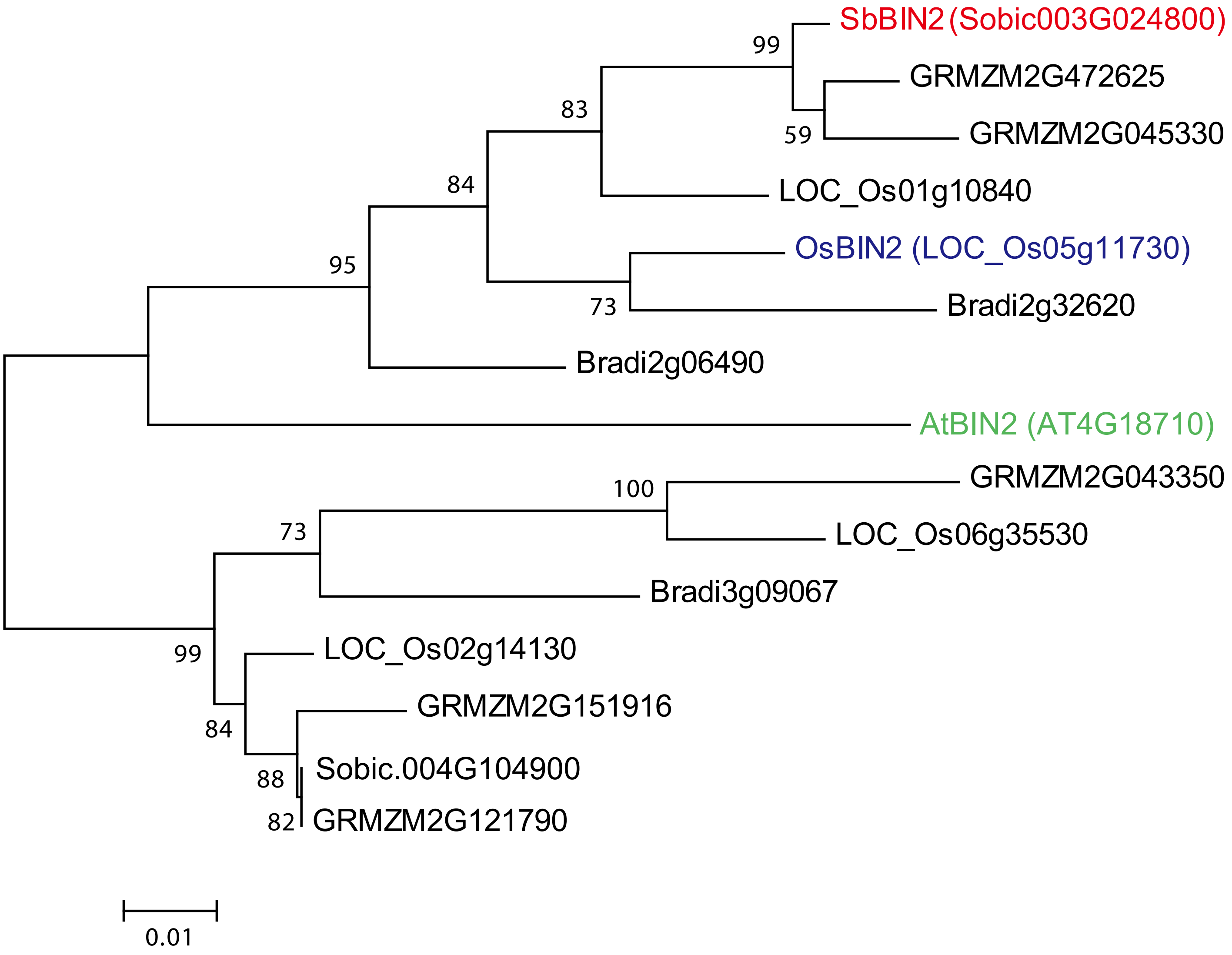

Fig. S4. Phylogenetic analysis of BIN2 homologs using the NJ method. SbBIN2 (Sobic.003G24800), OsBIN2 (LOC_Os05g11730), and AtBIN2 (AT4G18710) are shown in red, blue, and green, respectively. Bootstrap values were obtained by 100 bootstrap replicates.

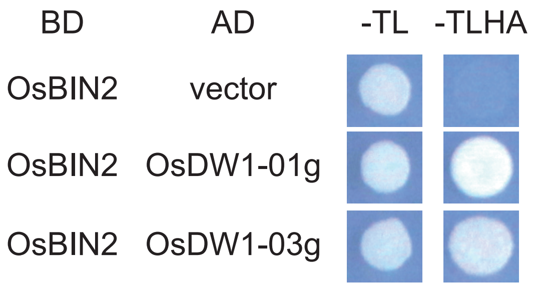

Fig. S5. Interaction of OsDW1-OsBIN2 in a Y2H assay. The growth of yeast strain AH109 transformants is shown. BIN2s were used as bait, and DW1s were used as prey. −TL, synthetic complete medium lacking Trp and Leu; −TLH A, synthetic complete medium lacking Trp, Leu, His, and Ade. OsDW1-01g and OsDW1-03g refer to LOC_Os01g01390 and LOC_Os03g16400, respectively.

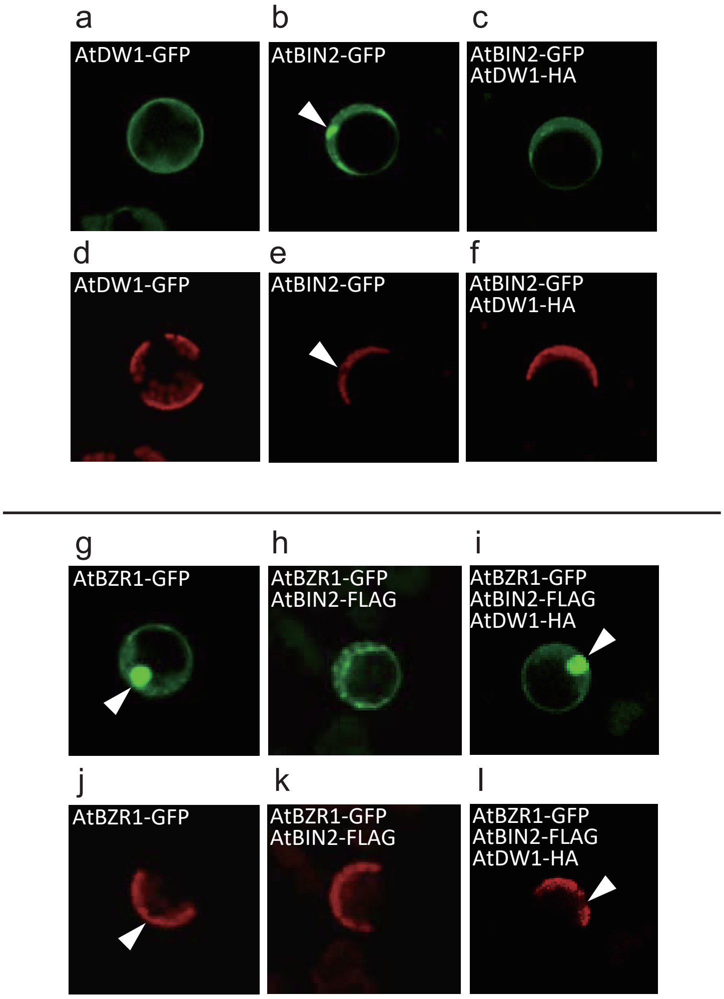

Fig. S6. Transient expression of AtDW1, AtBIN2, and AtBZR1 in *Arabidopsis* protoplasts. (a) AtDW1 localizes on the plasma membrane and in the cytosol. (b) AtBIN2 localizes to the nucleus and the cytosol. (c) Simultaneous expression of AtDW1and AtBIN2 inhibits nuclear localization of AtBIN2. (g) AtBZR1 localizes to the nucleus and the cytosol. (h) Simultaneous expression of AtBZR1 and AtBIN2 inhibits nuclear localization of AtBZR1. (i) AtDW1, AtBIN2, and AtBZR1 co-expression results in AtBZR1 localizing to the nucleus. *Arabidopsis* protoplasts were transformed with either AtBIN2-GFP, AtBZR1-GFP, AtDW1-GFP, and co-transformed with AtBIN2-FLAG, AtDW1-HA, or AtBIN2-FLAG/AtDW1-HA. (d-f, j-l) Chloroplast autofluorescence of *Arabidopsis* protoplasts are shown as the red channel.

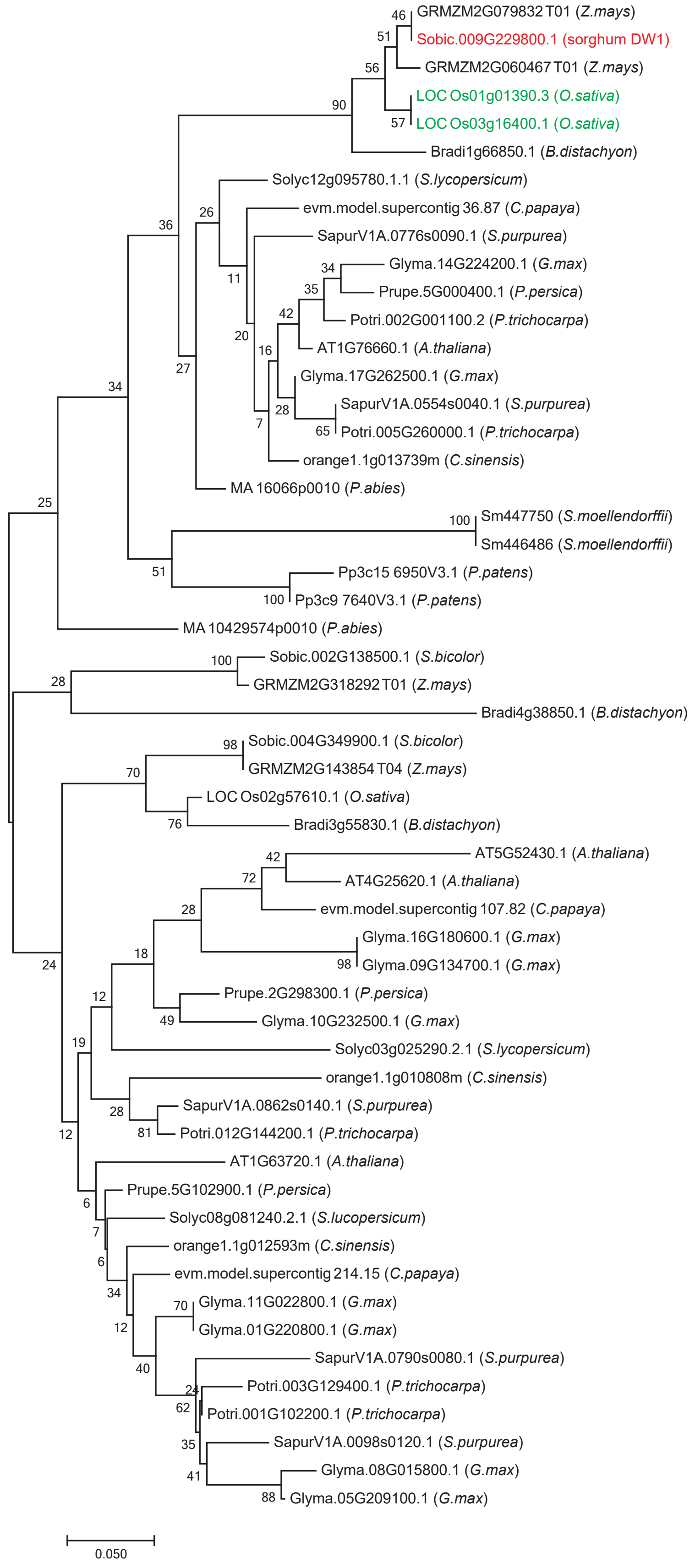

Fig. S7. Phylogenetic analysis of DW1 homologs using the neighbor-joining method. Bootstrap values were obtained by 100 bootstrap replicates.

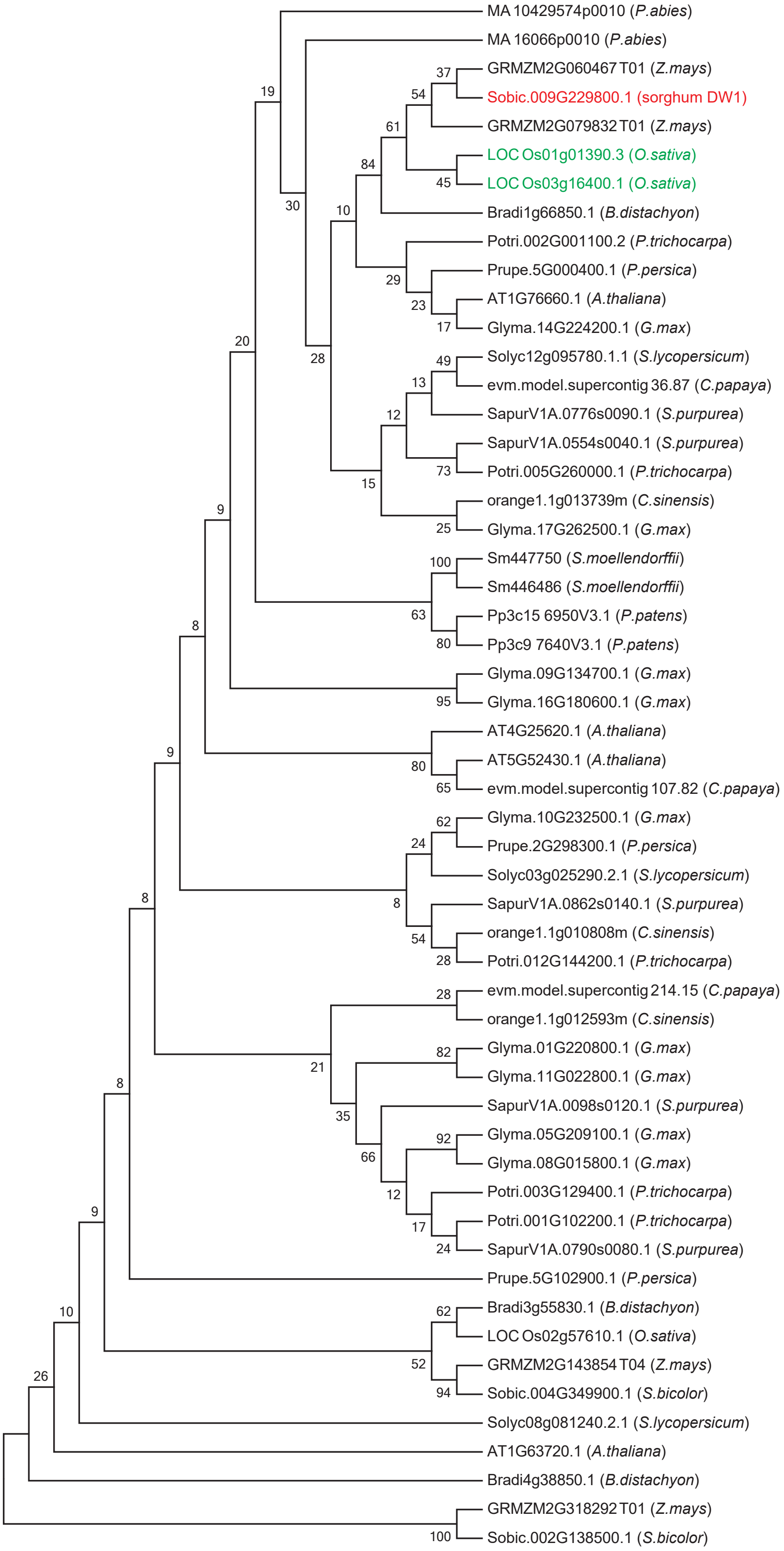

Fig. S8. Phylogenetic analysis of DW1 homologs using the maximum parsimony method. Bootstrap values were obtained by 100 bootstrap replicates.

Supplemental Table 1. Dw1 homologs used to create phylogenetic trees.

plant species	gene name	e-value[a]
**grass**		
S. bicolor	Sobic009G229800.1	0
	Sobic004G349900.1	7.30E-23
	Sobic002G138500.1	8.10E-09
O. sativa	LOC_Os03g16400.1	4.50E-175
	LOC_Os01g01390.3	7.60E-171
	LOC_Os02g57610.1	1.80E-20
B. distachyon	Bradi1g66850.1	0
	Bradi3g55830.1	2.90E-21
	Bradi4g38850.1	5.40E-07
Z. mays	GRMZM2G079832_T01	0
	GRMZM2G060467_T01	0
	GRMZM2G143854_T04	3.90E-22
	GRMZM2G318292_T01	5.60E-10
**dicotyledonous**		
A. thaliana	AT1G76660.1	2.20E-60
	AT1G63720.1	4.80E-31
	AT5G52430.1	1.60E-16
	AT4G25620.1	5.80E-07
G. max	Glyma.17G262500.1	3.60E-75
	Glyma.14G224200.1	2.70E-73
	Glyma.01G220800.1	1.80E-19
	Glyma.11G022800.1	5.80E-19
	Glyma.09G134700.1	8.20E-18
	Glyma.16G180600.1	3.00E-17
	Glyma.05G209100.1	5.20E-17
	Glyma.10G232500.1	1.10E-15
	Glyma.08G015800.1	8.80E-11
S. lycopersicum	Solyc12g095780.1.1	7.00E-56
	Solyc08g081240.2.1	1.80E-24
	Solyc03g025290.2.1	6.00E-20
P. persica	Prupe.5G000400.1	1.40E-70
	Prupe.5G102900.1	1.20E-67
	Prupe.2G298300.1	4.30E-20
C. sinensis	orange1.1g013739m	1.90E-74
	orange1.1g010808m	1.40E-22
	orange1.1g012593m	3.80E-20
C. papaya	evm.model.supercontig_36.87	1.10E-44
	evm.model.supercontig_214.15	1.30E-23
	evm.model.supercontig_107.82	3.10E-20
S. purpurea	SapurV1A.0776s0090.1	8.40E-73
	SapurV1A.0554s0040.1	1.50E-67
	SapurV1A.0862s0140.1	4.80E-20
	SapurV1A.0098s0120.1	1.60E-17
	SapurV1A.0790s0080.1	1.20E-15
P. trichocarpa	Potri.002G001100.2	1.40E-72
	Potri.005G260000.1	4.40E-69
	Potri.012G144200.1	1.70E-20
	Potri.001G102200.1	7.80E-18
	Potri.003G129400.1	1.10E-16
**gymnosperm**		
P. abies	MA_16066p0010	1.19E-53
	MA_10429574p0010	1.65E-23

**lycophyte**		
S. moellendorffii	Sm446486	5.40E-24
	Sm447750	1.20E-23

**bryophyte**		
P. patens	Pp3c9_7640V3.1	3.40E-19
	Pp3c15_6950V3.1	1.40E-18

[a.] tblastn was conducted using Sobic009G229800.1 (SbDW1) as query.

Supplemental Table 2. Details of primers used in this study

Primer name	Sequence (5' to 3')	Purpose
SbD2_RT_F	CTACGATGAGCCCTACAGGTTC	RT-PCR (Os01g0103800)
SbD2_RT_R	ACCAAGTGATGGAGAAAGATGG	
SbBR6ox_RT_F	TTATAGCGAGGATATAGGTATCATAGG	RT-PCR (Os03g0270700)
SbBR6ox_RT_R	TTCTGTTTAAATTTGAAAATTGATGG	
SbBRI1_RT_F	CTGCCCAGGGGTTAGTAGTG	RT-PCR
SbBRI1_RT_R	TGTACCATCAGAAGGGAATGC	
SbDW1_RT_F	TGCATTGAATTCGAGCTGTC	RT-PCR (SbDW1 BL treatment)
SbDW1_RT_R	ATTTCACTGCTCCTGCGAAC	
SbDW1_RT_F	GTCTTCAGTTGGGAGCAGCCCTGGCACCAG	RT-PCR (organ expression)
SbDW1_RT_R	ATTGGCACCAGATTGACCATTTCCACGAGC	
SbUbiquitin_RT_F	TGCAGTGGAGCGTCTTCTC	RT-PCR (sorghum ubiquitin)
SbUbiquitin_RT_R	CGTTAAGCCAAACACTCCTG	
SbDW1_AD_F	GCGGATCCATATGTCTTCAGTTGGG	Y2H
SbDW1_AD_R	GGCTCGAGTTAATGCAATTGTCTTGC	
SbBIN2_BD_F	GCGAATTCATGGAGGCGCCGCCGGGAC	Y2H
SbBIN2_BD_R	GCGTCGACCTAGCTCCCAACATGCCC	
OsDW1-01g_AD_F	GCGGATCCATATGGCGGCTACACCAG	Y2H (Os01g0103800)
OsDW1-01g_AD_R	GGCTCGAGTTACTGTAGTTGCCTTGC	
OsDW1-03g_AD_F	GCGGATCCATATGTTCGCTGTTGGGC	Y2H (Os03g0270700)
OsDW1-03g_AD_R	GGCTCGAGTTACTGTAGTTGCCTTGC	
OsBIN2_BD_F	GCGAATTCATGGACCAGCCGGCGCCG	Y2H
OsBIN2_BD_R	GCGTCGACTTAGCTCCCAGTATTGAAG	
AtDW1_AD_F	GCGAATTCATGGGCTCAGAGCAGGATC	Y2H
AtDW1_AD_R	GGCCCGGGTCAGGCTTTCCTGTGTCTGTTC	
AtBIN2_BD_F	GCGAATTCATGGGCTCAGAGCAGGATC	Y2H
AtBIN2_BD_R	GGCCCGGGTCAGGCTTTCCTGTGTCTGTTC	
SbDW1-GFP_F	CACCATGTCTTCAGTTGGGAGCAGCC	transient assay construction
SbDW1-GFP_R	ATGCAATTGTCTTGCCAATGCACTCC	
SbBIN2-GFP_F	TACAACTACATCTAGATGGAGGCGCCGCCGGGACC	transient assay construction
SbBIN2-GFP_R	TGCTCACCATACTAGTGCTCCCAACATGCCCGAAGT	
SbBZR1-GFP_F	TACAACTACATCTAGAATGACGTCGGGGGCGGCC	transient assay construction
SbBZR1-GFP_R	TGCTCACCATACTAGTCTTGGCGCCGACGCCAAG	
SbBIN2-FLAG_F	CACCATGGAGGCGCCGCCGGGACCGG	transient assay construction
SbBIN2-FLAG_R	GCTCCCAACATGCCCGAAGTTGACAC	
SbDW1-HA_F	GGCCAGTGCCAAGCTTTGCAGCGTGACCCGGTCG	transient assay construction
SbDW1-HA_R	CGTATGGGTAACTAGTATGCAATTGTCTTGCCAATG	
AtBIN2-GFP_F	TACAACTACATCTAGATGGCTGATGATAAGGAGAT	transient assay construction
AtBIN2-GFP_R	TGCTCACCATACTAGTAGTTCCAGATTGATTCAAGA	
AtBZR1-GFP_F	TACAACTACATCTAGAATGACTTCGGATGGAGCT	transient assay construction
AtBZR1-GFP_R	TGCTCACCATACTAGTACCACGAGCCTTCCCATT	
AtBIN2-FLAG_F	TACAACTACATCTAGAATGGCTGATGATAAGGAGAT	transient assay construction
AtBIN2-FLAG_R	CTTTGTAGTCACTAGTAGTTCCAGATTGATTCAAGAA	
AtDW1-HA_F	CGACTCTAGAGGATCCATGGGCTCAGAGCAGGAT	transient assay construction
AtDW1-HA_R	CGTATGGGTAACTAGTGGCTTTCCTGTGTCTGTTC	
AtDW1-GFP_F	TACAACTACATCTAGAATGGGCTCAGAGCAGGAT	transient assay construction
AtDW1-GFP_R	TGCTCACCATACTAGTGGCTTTCCTGTGTCTGTTC	
